# Host lipid sensing promotes invasion of cells with pathogenic *Salmonella*

**DOI:** 10.1038/s41598-018-33319-9

**Published:** 2018-10-19

**Authors:** Sonia Shivcharan, Jitender Yadav, Ayub Qadri

**Affiliations:** Hybridoma Laboratory, National Institute of Immunology, Aruna Asaf Ali Marg, New Delhi, 110067 India

## Abstract

Pathogenic *Salmonella* species initiate infection by invading non-phagocytic intestinal epithelial cells (IEC). This invasion is brought about by a number of *Salmonella* invasion promoting molecules (Sips) encoded by the *Salmonella* Pathogenicity Island - 1 (SPI-1). Intracellular delivery of some of these molecules also brings about caspase-1 – mediated pyroptotic cell death that contributes to pathogen clearance. These molecules are secreted and delivered inside cells upon contact of *Salmonella* with one or more host signals whose identity has not been established. We show that lysophosphatidylcholine (LPC) released following activation of caspase-1 in *Salmonella* – infected cells and abundant in plasma amplifies production of Sips from this pathogen and promotes its cellular invasion. LPC brings about adenylate cyclase and cAMP receptor protein (CRP) - dependent *de novo* synthesis of SipC that is accompanied by its translocation to bacterial cell surface and release into the outside milieu. Treatment of *Salmonella* with LPC produces sustained induction of SPI - 1 transcriptional regulator, hilA. Our findings reveal a novel host lipid sensing - driven regulatory mechanism for *Salmonella* invasion.

## Introduction

The establishment and outcome of infection with microbial pathogens involves an intimate cross-talk between the pathogen and the host. Bacterial pathogens have devised special strategies to invade host cells and produce a successful infection. Pathogenic *Salmonella* species invade non-phagocytic intestinal epithelial cells by delivering a specialized set of effectors through a sophisticated machinery comprising of the Type 3 secretion system (T3SS)^1^. The genes responsible for the T3SS clustered on the *Salmonella* pathogenicity island-1 (Spi-1) encode structural as well as secretory effectors (the invasion proteins) which play a central role in the pathogenesis of *Salmonella*^2^. T3SS-based invasosome is composed of a cytosolic basal body, a needle-complex traversing the bacterial membranes and protruding from the bacterial surface and a tip that is composed of translocases capable of forming pores in host cells^3^. The T3SS effectors SipB, SipC and SipD are required for intimate attachment of the bacterium to the epithelial cells while intracellularly delivered SipA, SipC, SopB and SopE act synergistically to promote formation of membrane ruffles at the point of bacterial contact and enable pathogen uptake by these cells^4,5^. SipA and SopE also induce activation of NF-κB and MAP-kinase pathways of intracellular signalling to bring about secretion of inflammatory cytokines and chemokines from epithelial cells^6^. Invasion of these cells also brings about caspase-1 – mediated cell extrusion that plays a critical role in clearing this pathogen from the gut^7^. This proinflammatory cell death is associated with release of eicosanoids and IL-18^8^. Contact with epithelial cells has been shown to transiently up-regulate the surface expression of the T3SS invasosome appendages by *Salmonella* in a translation-independent manner, and several environmental cues have been shown to modulate the expression of SPI-1^9–12^. However, the identity of the host signal(s) that induces expression and release of *Salmonella* effectors during invasion of cells with this pathogen has not been established. In the present study, we show that LPC, which is released upon activation of caspase-1 in *Salmonella* - infected cells, enhances production and release of invasion - promoting molecules from this pathogen thereby increasing its invasion ability. LPC mediates this amplification through adenylate cyclase and CRP – dependent signaling in *Salmonella*. Our findings reveal a previously unrecognized sensing mechanism that modulates invasion during infection with *Salmonella*.

## Results

### Encounter with host cells triggers release of Sips and enhances the ability of *Salmonella* to invade IECs

**Figure 1:**
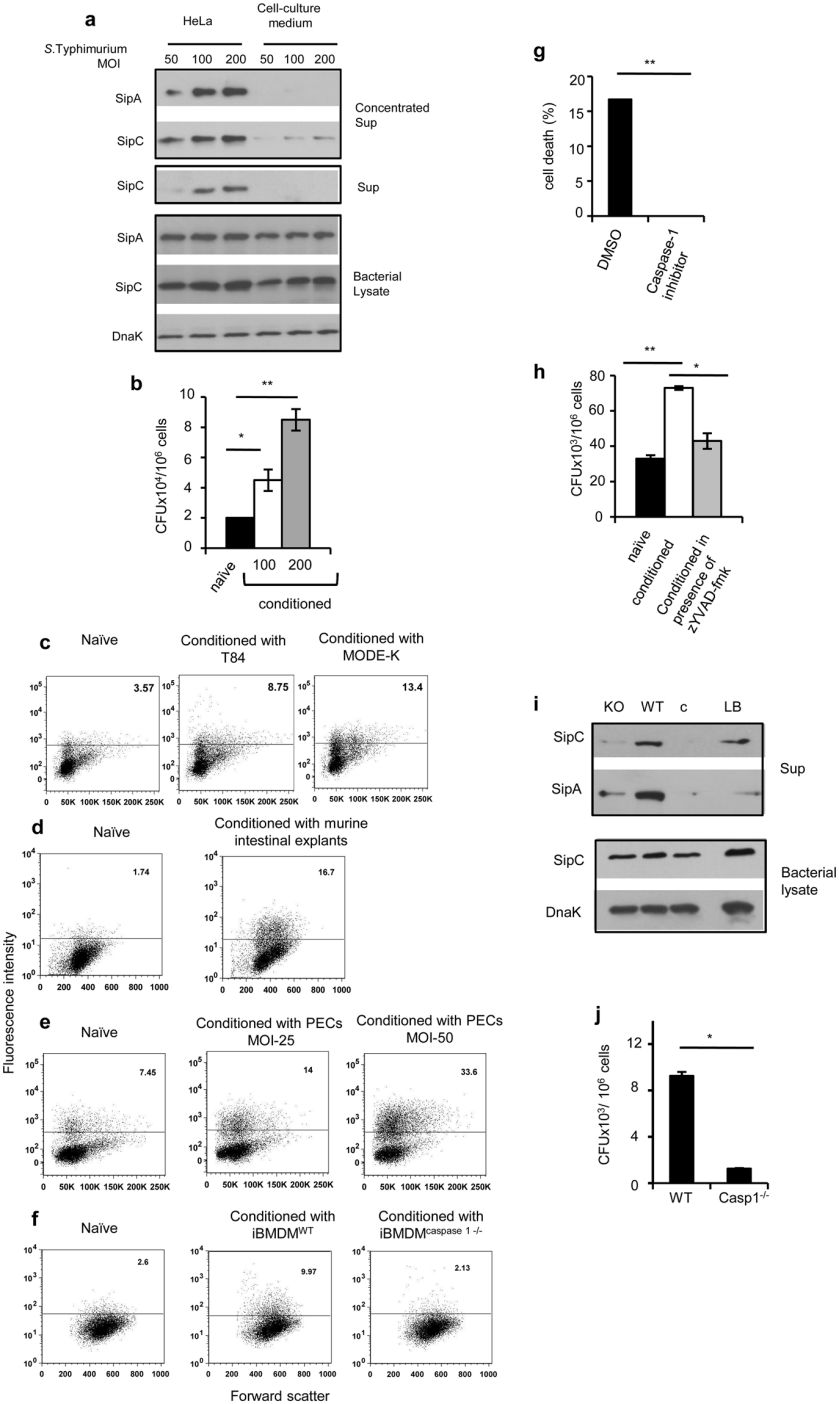
Caspase-1 – mediated cell death-dependent stimulus from host cells enhances invasion ability of pathogenic *Salmonella*. (**a**) SipA and SipC expression in cell-free supernatants (sup) and corresponding bacterial lysates from *S*. Typhimurium incubated for 1 h either with serum free cell culture medium (control) or with Hela cells at a pathogen to cell ratio of 50:1, 100:1 and 200:1 (conditioned). Concentrated supernatant represents TCA precipitated cell-free supernatants from bacteria cultured with serum-free medium or with Hela cells, and sup represents cell-free supernatants without concentration. DnaK was used as a loading control for cell lysates throughout the study. (**b**) Bacterial load in Hela cells infected for 1 h with *S*. Typhimurium (naïve or conditioned as indicated) at 50 multiplicity of infection (MOI). (**c–f**) Flow cytometric analysis of Hela cells infected at MOI 100 with GFP-expressing *S*. Typhimurium, naïve or conditioned for 1 h with human intestinal epithelial cell line T84 (**c**), murine intestinal epithelial cell line MODE-K at MOI 100 (**c**), ileal explants prepared from C57BL/6 mice (**d**), PECs (**e**) or with iBMDM lines (**f**). Numbers indicate percent cells containing GFP-positive *Salmonella*. (**g**) Percentage cell death in Hela infected with *S*. Typhimurium at MOI 100 in the presence of caspase-1 inhibitor (z-YVAD; 100 µM) or vehicle control (DMSO). (**h**) Intracellular bacterial load in Hela cells infected for 1 h at MOI 50 with *S*. Typhimurium, naïve or conditioned with Hela in the absence or presence of caspase-1 inhibitor. (**i**) SipA and SipC expression in cell-free supernatants from bacteria cultured in serum-free cell culture medium (**c**) or conditioned with iBMDMs, wild-type (WT) and caspase-1-deficient (KO). Sips in supernatants and lysates from bacteria grown overnight in LB are shown as positive control. Bacterial lysates were probed for DnaK and SipC. (**j**) Bacterial load in Hela cells infected at MOI 20 with bacteria recovered from WT or caspase-1 deficient iBMDMs after 6 h of infection. Error bars in (**b**,**g**,**h**,**j**) represent mean ± standard deviation (s.d.). Statistical significance was determined by two tailed student’s t-test. ^*^p < 0.05, ^**^p < 0.01. Immunoblot data within a marked box represents cropped images from one membrane probed with one or more antibodies. The full length immunoblots are presented in the supplementary figures with cropped regions marked by boxes.

To investigate the effect of host - sensing on bacterial invasion, we analyzed release of invasion-promoting molecules, SipA and SipC, in cell-free supernatants from co-cultures of *S*. Typhimurium with model epithelial cell line, Hela. These two molecules were released in the co-culture supernatant in an MOI-dependent fashion with SipC detectable more readily than SipA (Fig. 1a). Monoclonal antibodies generated against these two proteins were highly specific and did not bind any other *Salmonella* secretory protein (Supplementary Fig. S1). Bacteria derived from *Salmonella*-Hela co-cultures (referred to as “conditioned” *Salmonella*) were consistently more invasive as compared to bacteria incubated with serum-free cell culture medium alone (referred to as “naïve” *Salmonella*; Fig. 1b). This effect was also seen when Hela was infected with bacteria from co-cultures of *S*. Typhimurium with human intestinal colonic epithelial cell line T84, with murine small intestinal epithelial cell line, MODE-K (Fig. 1c) or with ileal intestinal explants prepared from C57BL/6 mice (Fig. 1d). The enhanced invasion ability was not observed only when Hela cells were used as hosts for invasion with conditioned bacteria; it was also evident when *Salmonella* conditioned with IEC lines (T84 and MODE-K) were used to infect these same cell lines (Supplementary Fig. S2). Importantly, co-culture of Hela with SipC deficient derivative of *S*. Typhimurium, which is poor in making intimate contact with cells and consequently deficient in invasion^5,13^, did not increase the invasion capability of this pathogen (Supplementary Fig. S3). This result suggested that efficient interaction of *Salmonella* with cells was required for generating the invasion-promoting host stimulus.

The ability of *S*. Typhimurium to invade cells also increased upon its interaction with resident peritoneal exudate cells (PECs) isolated from mice, immortalized bone marrow-derived macrophage cell line derived from wild-type (WT) C57BL/6 mice (iBMDM^wt^) but not with an immortalized BMDM line derived from caspase-1 deficient mice (iBMDM^casp1−/−^; Fig. 1e,f). As expected, infection with *S*. Typhimurium brought about cell death in iBMDM^wt^ and PECs but not in iBMDM^casp1−/−^ (Supplementary Fig. S4). This result suggested that the stimulus responsible for amplifying invasion ability of *Salmonella* might be generated through activation of caspase-1 in infected cells. This possibility was also supported in IECs when the first infection was carried out in presence of caspase-1 inhibitor zYVAD. This inhibitor reduced cell death produced by *Salmonella* (Fig. 1g). More importantly, *S*. Typhimurium taken from inhibitor-pretreated co-culture was less invasive as compared to bacteria taken from untreated co-cultures thereby establishing that even in IECs the stimulus responsible for enhancing invasion ability of *Salmonella* was generated upon caspase-1 activation (Fig. 1h). Consistent with these results, infection of peritoneal macrophages and iBMDM^wt^ but not iBMDM^casp1−/−^ with *S*. Typhimurium released more SipA and SipC in the extracellular medium clearly establishing the ability of caspase-1–generated stimulus to promote release of invasion-promoting molecules from *Salmonella* (Fig. 1i, Supplementary Fig. S4). Significantly, the increased invasion ability was also imprinted on intracellular *Salmonella* isolated from iBMDM^wt^ (Fig. 1j).

**Figure 2:**
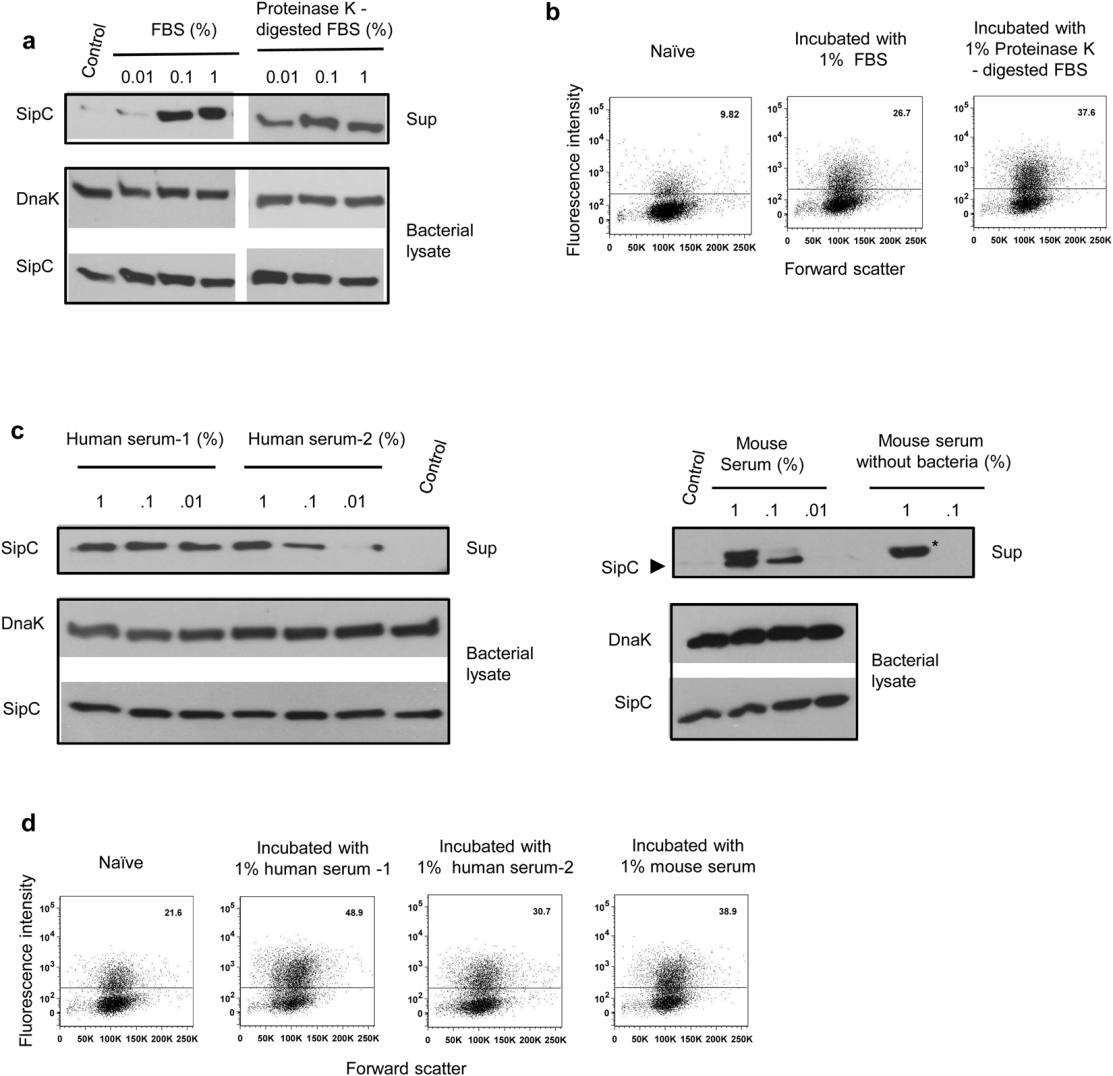
Serum borne lipids induce secretion of SipC and increase invasion capability of *Salmonella*. (**a**) Secretion of SipC in the supernatants of *S*. Typhimurium incubated with different concentrations of undigested or Proteinase-K digested FBS. Bacterial lysates were probed for SipC and DnaK antibodies. (**b**) Flow cytometric analysis of Hela cells infected with GFP-expressing *S*. Typhimurium stimulated with 1% Proteinase K-digested or undigested FBS. (**c**) Release of SipC by *S*. Typhimurium incubated with increasing concentrations of human or mice sera for 1 h at 37 °C. Bacterial lysates were probed for SipC and DnaK. A non-specific band of higher molecular weight (denoted by asterisk) in addition to SipC (denoted by filled black arrowhead) was also observed in the supernatant with higher concentration of mouse serum. (**d**) Flow cytometric analysis of Hela cells infected with GFP-expressing *S*. Typhimurium stimulated with human (two healthy donors) or mice sera for 1 h at 37 °C. Numbers indicate percent cells containing GFP positive bacteria. Data are representative of 2–3 independent experiments. Immunoblot data within a marked box represents cropped images from one membrane probed with one or more antibodies. The full length immunoblots are presented in the supplementary figures with cropped regions marked by boxes.

In the course of this analysis, increased secretion of SipC accompanied by increase in the invasion ability of *S*. Typhimurium was also seen upon incubation of bacteria with fetal bovine serum (FBS) (Fig. 2a,b; Supplementary Fig. S5). This activity was not abrogated upon digestion of FBS with Proteinase-K, which suggested that the factor(s) responsible for this activity might be lipid in nature (Fig. 2a,b; Supplementary Figs S5 and S6). Further experiments revealed that this activity was not restricted to FBS, it was also present in mouse and human sera (Fig. 2c). Sera from two apparently healthy subjects differed in their ability to bring about release of SipC from *Salmonella*. This difference correlated well with their ability to promote invasion of *Salmonella* (Fig. 2d).

### Lysophospholipids bring about induction and release of *Salmonella* invasion – promoting molecules

**Figure 3:**
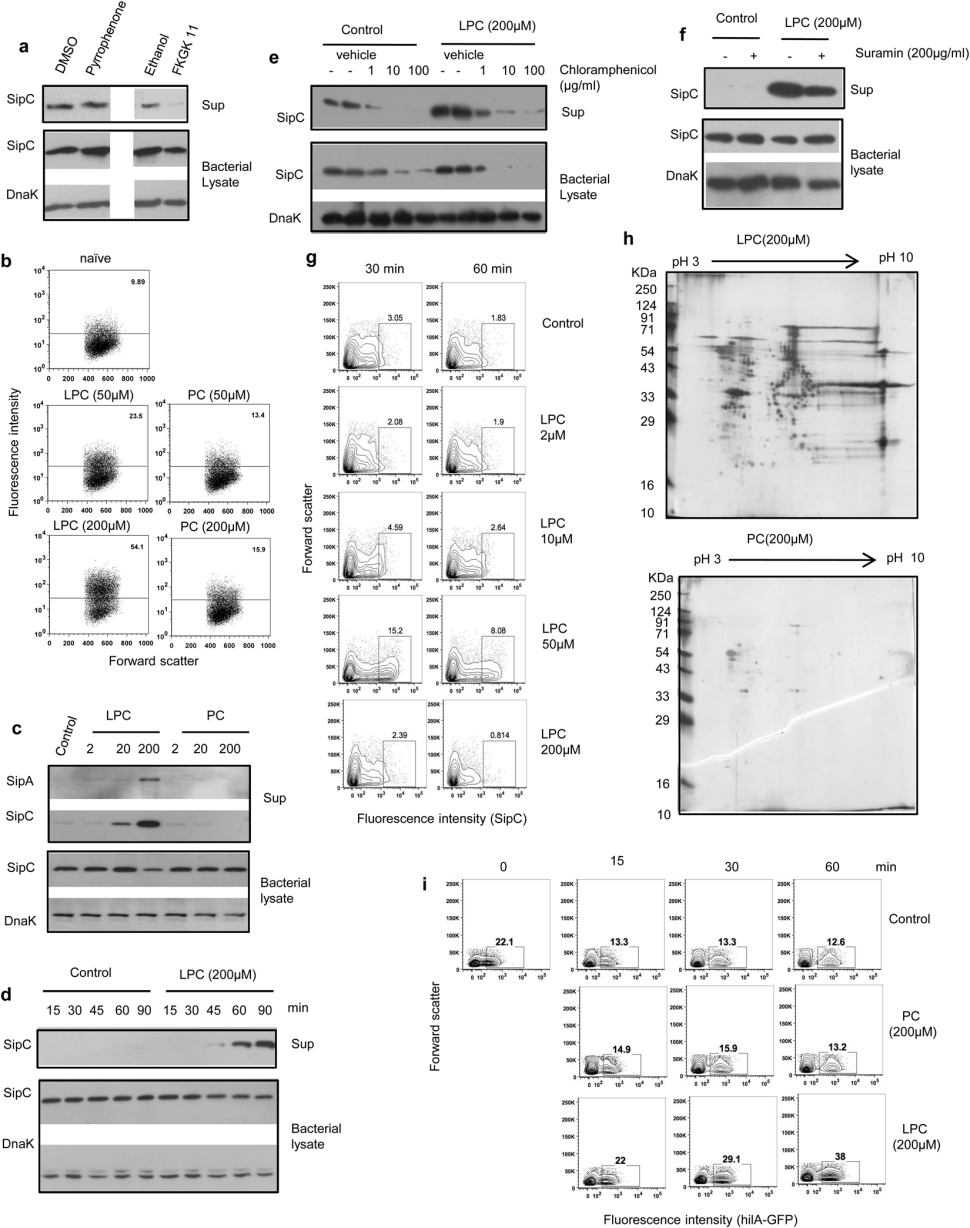
Activation with lysophosphatidylcholine (LPC) induces expression and release of *Salmonella* invasion-promoting molecule*s*, and increases invasion ability of *Salmonella*. (**a**) SipC expression in cell-free supernatants from *S*. Typhimurium co-cultured with Hela cells in the absence or presence of cPLA2 inhibitor, Pyrrophenone (0.5 µM) or iPLA2 inhibitor, FKGK11 (30 µM) or their respective vehicle controls. Bacterial lysates were also probed for SipC and Dnak. (**b**) Flow cytometric analysis of Hela cells infected at MOI 100 with GFP-labeled *S*. Typhimurium stimulated with indicated concentrations of LPC or PC for 1 h. Numbers indicate percent cells containing GFP-positive *Salmonella*. (**c**) Expression of SipA, SipC and DnaK in cell-free supernatants and corresponding bacterial lysates derived from *S*. Typhimurium incubated with increasing concentrations of LPC or PC. (**d**) Expression of SipC in the supernatants and lysates of *S*. Typhimurium cultured in absence or presence of LPC (200 μM) for increasing time durations. Bacterial lysates were also probed for DnaK. (**e**,**f**) The expression of SipC in supernatants and lysates of *S*. Typhimurium stimulated with LPC (200 μM) in presence of indicated concentrations of Chloramphenicol or vehicle control (**e**), or in the presence of suramin (200 μg/ml) or vehicle control (**f**). (**g**) Flow cytometric analysis of expression of SipC on the surface of *S*. Typhimurium following activation with different concentrations of LPC for 30 and 60 minutes. Numbers indicate percent SipC-expressing bacteria. (**h**) 10^8^ *S*. Typhimurium were stimulated with LPC or PC (200 µM) and after 1 h, bacteria-free supernatants were subjected to 2D gel electrophoresis. The spots in the gel were visualized by silver staining. (**i**) Flow cytometric analysis of GFP expression in *S*. Typhimurium expressing GFP driven by Hil-A promoter upon treatment with LPC or PC (200 µM) for different time durations. Data are representative of 3 independent experiments. Immunoblot data within a marked box represents cropped images from one membrane probed with one or more antibodies. The full length immunoblots are presented in the Supplementary Figures with cropped regions marked by boxes.

Our previous study had shown that sensing of lysophospholipids including LPC produced by IECs can bring about production of proinflammatory flagellin from *Salmonella*^14^. Later studies by Von-Moltke *et al*. and Rauch *et al*. have revealed that pyroptotic cell death produced by caspase-1 activation following infection with pathogenic *Salmonella* is associated with a lipid storm releasing eicosanoids including prostaglandins^8,15^. Our own analysis showed that pyroptosis also releases LPC (unpublished data). To explore if lysophospholipids such as LPC, which might be released during infection of epithelial cells with *Salmonella*, are involved in activating release of SipC from the pathogen, cells were treated with inhibitors of calcium independent phospholipase-A2 (iPLA2) and calcium dependent phosphoplipase-A2 (cPLA2) before infecting with *S*. Typhimurium. Inhibition of iPLA2 but not cPLA2 resulted in significant reduction in the release of SipC in bacteria-cell co-cultures strongly indicating a role for lysophospholipids in bringing about release of Sips from *Salmonella* (Fig. 3a). Based on this result, subsequent experiments were carried out to study the role of LPC in modulating invasion of cells with *Salmonella*. Treatment with LPC increased the invasion efficacy of *Salmonella* in a dose-dependent fashion (Fig. 3b). This effect was specific to LPC as similar treatment with PC did not significantly alter the invasion ability of *Salmonella* (Fig. 3b). This modulation was not restricted to Hela as LPC-treated *Salmonella* were also more invasive with murine intestinal cell line MODE-K and human intestinal cell line, T84 (Supplementary Fig.S7). LPC increased release of Sips A and C in a dose - dependent manner without affecting bacterial replication (Fig. 3c and Supplementary Fig. S7). Similar increase was not seen with PC (Fig. 3c). As the expression of SipA and SipC is co-regulated, further experiments were carried out by analyzing expression of SipC in detail. Treatment with LPC induced expression of SipC in *Salmonella* in a time - dependent fashion (Fig. 3d). LPC did not simply bring about release of pre-existing SipC; it induced its *de novo* synthesis that was accompanied by concomitant release (Fig. 3d). Consistent with this mechanism, the induction of SipC was reduced when bacteria were pre-treated with chloramphenicol before stimulation with LPC (Fig. 3e). In fact, the expression of SipC was also reduced in unstimulated bacteria pre-treated with chloramphenicol (Fig. 3e). Chloramphenicol treatment did not affect expression of Dnak (Fig. 3e). When stimulation of bacteria with LPC was carried out in presence of suramin, which is a known inhibitor of bacterial ATPase, the release of SipC was reduced and more SipC was retained inside bacteria (Fig. 3f, Nautiyal *et al*., 2014)^16^. The expression of SipC on the surface of bacteria was also increased as determined by flow cytometry (Fig. 3g). SipC was detected only on a subset of LPC-stimulated *Salmonella* indicating heterogeneity in the response of this pathogen to this lipid (Fig. 3g). This heterogeneity might have implications for the outcome of infection as translocation of SipC to the surface is essential for attachment of *Salmonella* to host cells^5^. Strikingly, unlike progressive time-dependent increase in SipC expression in the supernatant following activation of bacteria with increasing concentrations of LPC (at least up to 200 μM), the expression of cell surface associated SipC increased upon treatment with 50 µM LPC and dropped close to basal levels with 200 µM LPC (Fig. 3g). Also, while the pattern of expression showed a similar trend after 60 minutes of LPC treatment, the level of SipC expression on the surface was lesser than what was observed at 30 minutes with all the concentrations of LPC tested (Fig. 3g). The reasons for these differences are not clear at the moment. It looks likely that SipC is first decorated on the surface and later released into extracellular medium, and more potent stimulation promotes the release process. Further investigations on this aspect should offer new insights into the process of cellular invasion with *Salmonella*.

Analysis of cell free supernatants from LPC-activated *S*. Typhimurium by two dimensional gel electrophoresis revealed that the effect of this lipid was not confined to SipA and SipC; LPC induced release of a large number of proteins from *Salmonella* (Fig. 3h). On the other hand, *Salmonella* treated with PC released a very small number of proteins (Fig. 3h). As the expression of invasion-regulating effectors encoded by SPI-1 is primarily controlled by the transcriptional regulator hilA, we studied induction of this molecule in LPC-treated bacteria^17^. Treatment with LPC produced sustained induction of hilA promoter – driven GFP expression in *Salmonella* (Fig. 3i)^18^. In contrast, untreated and PC - treated *Salmonella* showed a steady decline in the constitutive expression of hilA-dependent GFP (Fig. 3i). The induction of SipC in response to stimulation with LPC was not restricted to *S*. Typhimurium; it was readily seen with *S*. Typhi and *S*. Enteritidis as well (data not shown).

### LPC – triggered production of SipC from *Salmonella* is regulated by cAMP and CRP

**Figure 4:**
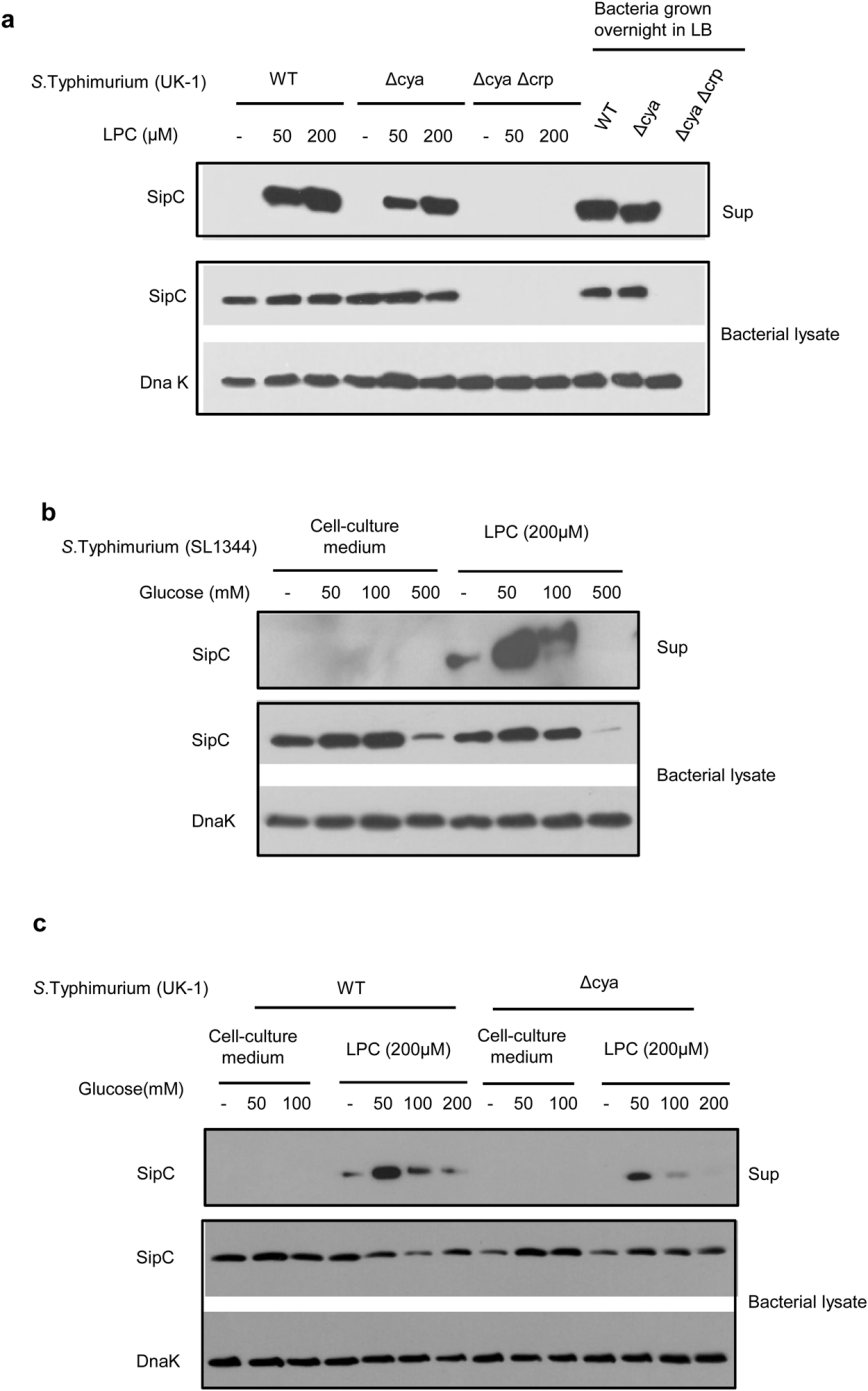
LPC activates release of SipC from *Salmonella* through cAMP-CRP dependent signaling pathway. (**a**) SipC levels were analyzed in the culture supernatants of Wild-type *S*. Typhimurium (UK-1 strain), its adenylate cyclase knock-out derivative (Δcya-27) and adenylate cyclase – CRP double knock-out strain (Δcya-Δcrp), treated with different concentrations of LPC. Expression levels from bacteria grown overnight in LB are shown as positive controls. Bacterial lysates were also probed for SipC and DnaK. (**b**) SipC levels were determined in (**b**) *S*. Typhimurium SL 1344 and (**c**) *S*. Typhimurium UK-1 and its Δcya-27 derivative stimulated with LPC (200 µM) in presence of increasing concentrations of glucose. Bacterial lysates were probed for SipC and DnaK. Data are representative of two independent experiments. Immunoblot data within a marked box represents cropped images from one membrane probed with one or more antibodies. The full length immunoblots are presented in the supplementary figures with cropped regions marked by boxes.

To understand the mechanism by which LPC brings about induction and release of SipC, we analyzed the role of cyclic AMP (cAMP) and cAMP receptor protein (CRP), which are known to regulate expression of SPI-1^19^, in this phenomenon. *S*. Typhimurium strain lacking adenylate cyclase (Δcya) produced less SipC as compared to the wild-type (WT) strain upon treatment with LPC (Fig. 4a). On the other hand, *S*. Typhimurium lacking adenylate cyclase as well as cAMP receptor protein (ΔcyaΔcrp) did not show any detectable SipC (Fig. 4a). In fact, this double mutant did not show constitutive expression of SipC, which suggested that CRP might be playing a critical role in regulating expression of Sips independent of cAMP^19^ (Fig. 4a). The inability of these mutant strains to respond to LPC might have also contributed to their attenuation^19,20^. The role of cAMP and CRP was also suggested by reduced expression of SipC in bacteria activated with LPC in presence of high amounts of glucose that is known to not only reduce levels of cAMP through catabolite repression but also down-regulate mRNA levels of CRP in bacteria lacking cAMP (Fig. 4b,c)^21,22^.

## Discussion

Pathogenic *Salmonella* enters non-phagocytic intestinal epithelial cells by delivering a complex set of effectors including Sips into the host cell. These molecules orchestrate actin cytoskeletal rearrangements that bring about membrane ruffling and enable bacterial invasion^23^. The Sips along with other bacterial effectors such as flagellin and LPS also activate the inflammasomes resulting in caspase-1 activation that mediates pyroptotic cell death accompanied by release of IL-1β, IL-18 and eicosanoids^8,15,24–28^. Pyroptosis plays a vital role in innate immunity against *Salmonella* in models of experimental infection^7,29,30^. Sips are released from bacteria and delivered into IECs following contact with host signal(s) whose identity has not been well established. In this study, we demonstrate that *Salmonella* senses LPC released during caspase-1 – mediated pyroptotic cell death, and this sensing brings about release of Sips and enhances the ability of the pathogen to invade cells. LPC upregulates expression and release of Sips through an adenylate cyclase-dependent signaling pathway that produces sustained induction of hilA, one of the major transcriptional regulators of Sips. The ability of IECs to activate release of SipC from *Salmonella* was found to be dependent on iPLA2. The latter has been previously shown to get activated during infection with *Salmonella* and bring about transmigration of neutrophils^31^. However, the mechanism by which *Salmonella* infection activates iPLA2 is not clear at the moment. One likely candidate is caspase-3 which gets activated during infection of IECs as well as macrophages with *Salmonella*, and is known to activate iPLA2 by cleaving its c-terminal ankyrin domain^32–35^. In IECs, caspase-3 is activated by SipA which interestingly also becomes one of its targets^34^. Irrespective of the exact mechanism by which *Salmonella* modulates levels of LPC, our results suggest that generation of this lipid early on from infected cells and its release from pyroptotic cells would serve as a stimulus for enhancing invasion ability of intracellular as well as extracellular *Salmonella*. In this context, recent studies have indicated that a subpopulation of *Salmonella* escapes the SCV (*Salmonella* containing vacuole) and replicates in the cytosol to produce a hyper-invasive and hyper-motile bacterial population, which serves as a reservoir for dissemination of the pathogen^36,37^. It seems very likely from our data that LPC produced as a result of caspase activation in the cytosol contributes to generation of this population. This lipid is present in abundance in plasma and could therefore serve as a physiological cue for a hyperinvasive phenotype during dissemination of *Salmonella* in blood.

LPC plays an important role in various cellular functions through activation of G-protein coupled receptors^38–40^. We have previously shown that it can bring about induction and release of flagellin from *Salmonella*^14^. Coupled with that study, we would like to propose that pyroptotic cell death early on during infection with *Salmonella* not only contributes to bacterial clearance but it also serves as an amplification loop for invasion as well as inflammation that enables the pathogen to infect larger repertoire of cells and establish its infection. However, as the infection progresses and *Salmonella* breaches the gut it switches to an SPI-1^low^ flagellin^low^ phenotype^41,42^. The exact mechanism of this switch has not been established. PLA2 levels have been shown to be elevated early on and decrease gradually and significantly with the progression of infection during typhoid fever^43^. More recent studies have revealed that LPC levels in plasma and in macrophages are considerably reduced during sepsis and infection with *Plasmodium* and several other pathogens in mice as well as humans^44^. Therefore, while induction and release of LPC during early stages of infection would promote invasion with *Salmonella*, limited availability of this lipid at later stages of infection could contribute to SPI-1^low^ flagellin^low^ phenotype that would enable *Salmonella* to avoid host sensing at systemic sites^41^. Taken together, our findings identify LPC as a key component of a novel regulatory mechanism for regulation of cellular invasion with pathogenic *Salmonella*.

## Materials and Methods

### Cell lines, bacterial strains and other reagents

The human colonic epithelial cell line T-84 and human cervical epithelial cell line, Hela were obtained from the American Type Culture Collection (ATCC). Murine small intestinal epithelial cell line, MODE-K, was kindly provided by Dominique Kaiserlian, INSERM-U851, France, and immortalized bone marrow-derived–macrophage lines (iBMDM) derived from wild-type (WT) and caspase-1 deficient mice were given by Eicke Latz, University of Bonn, Germany. *Salmonella enterica* serovar Typhimurium SL1344 (*S*. Typhimurium) was provided by Emmanuelle Charpentier, University of Vienna, Austria, GFP-expressing and SipC deficient *S*. Typhimurium strains by Amitabha Mukhopadhyay, National Institute of Immunology, New Delhi. *S*. Typhimurium SL1344 carrying GFP driven by Hil-A promoter (*S*. Typhimurium Hil-A) by Brett Finlay, University of British Columbia, Vancouver, Canada, and *S*. Typhimurium UK-1 strain and its adenylate cyclase deficient (Δcya) and adenylate cyclase and–cAMP receptor protein deficient (Δcya–Δcrp) derivatives were made available by Roy Curtiss Jr. (Arizona State University, Phoenix, Arizona). *S*.Typhi (Vi negative isolate) was provided by Geeta Mehta (Lady Hardinge Medical College, New Delhi, India) and *S*. Enteritidis was obtained from Mrutyunjay Suar, Kalinga Institute of Industrial Technology, Bhubaneswar, India. Lysophophatidylcholine (LPC) and phosphatidylcholine (PC) were obtained from Avanti Polar Lipids; polyclonal rabbit antiserum to *S*. Typhimurium flagellin from BD-Difco Laboratories, anti-DnaK antibody was from Enzo Life Sciences, cPLA2 and iPLA2 inhibitors, Pyrrophenone & FKGK-11 respectively, were from Cayman Laboratories, caspase-1 specific inhibitor zYVAD was from Calbiochem. Chloramphenicol, suramin and D-glucose were obtained from Sigma-Aldrich. Iso-electric focusing (IEF) strips for 2-D gel electrophoresis were purchased from GE Life sciences. Monoclonal antibodies against SipA and SipC were generated as described previously^45^.

### Cell and bacterial culture

T84 and MODE-K were grown in RPMI-1640 supplemented with 10% heat-inactivated fetal bovine serum (FBS; RPMI-10) in a humidified atmosphere at 37 °C with 5% CO_2_. Hela was cultured in DMEM supplemented with 10% FBS (DMEM-10). *S*. Typhimurium was grown in Luria Bertani (LB) broth supplemented with streptomycin (100 µg/ml for SL1344) or appropriate antibiotics (for other strains) at 37 °C with shaking (220 rpm).

### Conditioning of *Salmonella* with host stimulus

To investigate the effect of host-sensing on invasion ability, *S*. Typhimurium were either left in cell culture medium or co-cultured with epithelial cell lines, 1 cm long ileal explants derived from C57BL/6 mice, peritoneal exudate cells (PECs) isolated from mice or iBMDM for 1–2 h at 37 °C. Bacteria taken from cell culture medium alone (referred to as ‘naive’ throughout the study) and those from co-cultures (referred to as ‘conditioned’ throughout the study) were washed with serum-free cell culture medium, resuspended in this medium and used to infect fresh Hela monolayer (unless otherwise mentioned) for 1 h at 37 °C (Bacterial number was determined by measuring optical density at 630 nm). In some experiments, Hela cells were pretreated with caspase-1 inhibitor (zYVAD, 100 µM) half an hour before and during their first interaction with *Salmonella* (conditioning). To analyze the role of phospholipases, Hela cells were pre-incubated with Pyrrophenone (0.5 µM), FKGK11 (30 µM) or respective vehicle control overnight and during infection with *Salmonella*.

The role of caspase-1 in modulating the invasion ability of intracellular *Salmonella* was examined by infecting Hela with bacteria isolated from WT and caspase-1 deficient iBMDM lines. All experiments involving mice and human subjects were done in accordance with guidelines provided by the Institutional Animal Ethics Committee and the Institutional Human Ethics Committee of the National Institute of Immunology.

### Bacterial invasion

Hela cells suspended in complete medium were seeded at a density of 2 × 10^5^ cells per well in a 24-well plate one night prior to infection. Cells were washed 3 times with serum-free medium and infected with *S*. Typhimurium (in some experiments GFP-expressing strain was used for assessing invasion) at different MOIs for 1 h at 37 °C. Cells were washed to remove extracellular bacteria and cultured in complete medium supplemented with gentamycin (100 µg/ml). After 1 h, gentamycin was thoroughly washed off and cells were lysed with 0.1% Triton-X-100 in PBS. Cell lysates were plated on *Salmonella-Shigella* agar plates and the number of colonies was enumerated after overnight incubation at 37 °C. In experiments where GFP-expressing *Salmonella* was used, infected cells were washed to remove extracellular bacteria and treated with EDTA to get them off the plate. After washing with PBS, cells were fixed with 4% para-formaldehyde for 10 minutes and analyzed in a flow cytometer (BD FACS Verse). Data were plotted using FlowJo software.

### Determination of cell death

Cell death produced by *Salmonella* infection was determined by analyzing release of Lactate dehydrogenase (LDH) using the CytoTox 96 Non-radioactive Cytotoxicity Assay according to the manufacturer’s (Promega) instructions. The degree of death was calculated as follows:

Percent death (infected sample) = [LDH released from infected sample − spontaneous release (uninfected sample)] total LDH X100. LDH obtained from cells upon lysis with 1% TritonX-100 was taken as 100% death.

### Analysis of expression of Sips in *Salmonella*

Bacteria were grown in LB medium, washed with serum-free DMEM and resuspended in serum-free medium. Cells were infected with these bacteria at different MOIs for 1–2 h following which cell-free supernatants were collected, passed through a 0.22 μ membrane and subjected to western blot analysis with antibodies specific to SipA and SipC. In some experiments, cell-free supernatants were enriched for bacterial effectors by precipitation with trichloroacetic acid as described by Zierler and Galan except that precipitation was carried out overnight at 4 °C^9^. For stimulation with lipids, 10^8^ bacteria were incubated in 1 ml serum-free medium in the absence or presence of LPC or PC at 37 °C. Bacterial suspension was centrifuged at 8000 × g and the supernatant was filtered through a 0.22 μ membrane. Bacterial pellet and cell-free supernatants were boiled with Laemmli sample buffer. The samples were resolved by discontinuous sodium-dodecyl sulfate-polyacrylamide gel electrophoresis and transferred to a nitrocellulose membrane. The membrane was blocked with 5% non-fat milk in PBS and incubated with monoclonal antibodies against SipA and SipC, and anti-DnaK antibodies for 2 h at room temperature, followed by HRP-labeled anti-mouse Ig antibody. The blot was washed and bands were visualized using Enhanced Chemiluminescence Reagent (Amersham Pharmacia Biotech). In some experiments, bacteria were incubated with Chloramphenicol (1–100 µg/ml) for 30 minutes before incubating with LPC. Activation of *Salmonella* with LPC was also carried out in presence of bacterial ATPase inhibitor, suramin. The role of cAMP was analyzed by stimulating bacteria with LPC in presence of glucose (50–500 mM).

*Salmonella* were also stimulated for 1 h at 37 °C with fetal bovine serum, sera obtained from healthy human subjects or from normal C57BL/6 mice. The role of lipids present in serum in modulating bacterial invasion was examined by subjecting FBS to digestion with Proteinase K (100 µg/ml) followed by heat inactivation (80 °C for 50 minutes) and then using it to activate bacteria (Fig. 2b). The release of Sips in cell-free supernatants from these stimulations was analyzed by western blotting with specific antibodies as described above.

### 2D gel electrophoresis of *Salmonella* secretory proteins

10^8^ *S*. Typhimurium were stimulated with LPC or PC (200 µM) and after 1 h, bacteria-free supernatant passed through 0.22 µ membrane was precipitated with trichloroacetic acid. 2 D sample buffer was added to the precipitate and iso-electric focusing was carried out according to the protocol described by Adams and Gallagher (2004)^46^ using GE IPGphor device. After iso-electric focusing, the strip was placed on top of SDS-polyacrylamide gel and subjected to electrophoresis in the second dimension at 40 mA for 4 h. The spots in the 2D gel were visualized by silver staining as described by Sasse and Gallagher^47^.

### Analysis of Sip C and Hil A expression in *Salmonella* by flow cytometry

To analyze translocation of SipC to the surface of bacteria, 100 × 10^6^ *S*. Typhimurium were stimulated with different concentrations of LPC or PC for 30 and 60 minutes. After stimulation, bacteria were pelleted down, fixed with 4% PFA for 10 minutes. Bacteria were washed and incubated with monoclonal anti-SipC antibody for an hour followed by PE-labeled anti-mouse Ig antibody (Jackson). After washing, bacteria were analyzed by flow cytometry (BD FACS Verse).

*S*. Typhimurium strain containing GFP gene driven by Hil-A promoter was stimulated with LPC or PC (200 µM) for different time points. Bacteria were washed, fixed with 4% PFA and analyzed by flow cytometry.

### Statistical analysis

Data was analyzed using Microsoft Excel software. Error bars represent mean ± standard deviation. p values were calculated using a two-tailed Student’s *t*-test. A p value of less than 0.05 was considered statistically significant.

## Electronic supplementary material

Supplementary material

## Data Availability

All data discussed in this report is available upon request.
