# Supplementary material for: Host lipid sensing promotes invasion of cells with pathogenic Salmonella
Source: Sci Rep. 2018 Oct 19;8:15501. doi: 10.1038/s41598-018-33319-9 (PMC6195605; doi:10.1038/s41598-018-33319-9)
Supplement: Supplementary file 1 — Supplementary material [file 41598_2018_33319_MOESM1_ESM.pdf]

Supplementary figures

Host lipid sensing promotes invasion of cells with pathogenic *Salmonella*

Sonia Shivcharan[1,2], Jitender Yadav[1,2] and Ayub Qadri[1,*]

[1]Hybridoma Laboratory, National Institute of Immunology, Aruna Asaf Ali Marg, New Delhi-110067, India. [2]These authors contributed equally to this work.

[*]**Corresponding author**
Ayub Qadri, National Institute of Immunology, Aruna Asaf Ali Marg,
New Delhi – 110067, India.
Telephone: 91-11-26703733; Fax 91-11-26742125; e-mail: ayub@nii.ac.in

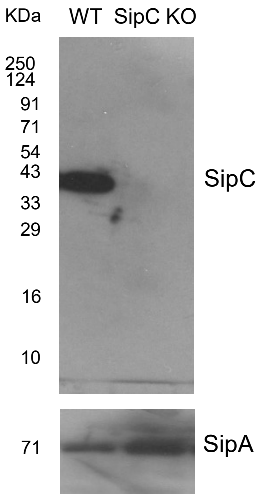

Supplementary Figure S1, Characterization of monoclonal antibodies. Immunoblot for SipC and SipA in bacteria - free culture supernatants prepared from *S.* Typhimurium (SL1344,WT) and its SipC deficient derivative (SipC KO) grown overnight in LB medium.

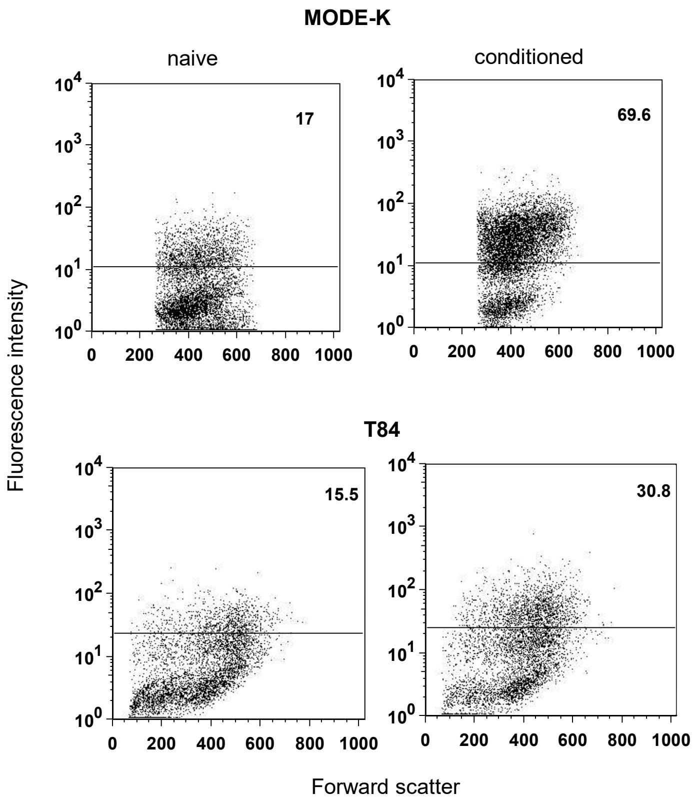

Supplementary Figure S2, Interaction with IECs enhances the invasion capability of *Salmonella.* Flow cytometric analysis of IEC lines, MODE-K and T84, infected for 1 h at 37°C with GFP-labeled *S*. Typhimurium (100 MOI), which had been conditioned for 1 h with MODE-K and T84 respectively. Numbers indicate percent cells containing GFP-positive bacteria. Data is representative of two independent experiments.

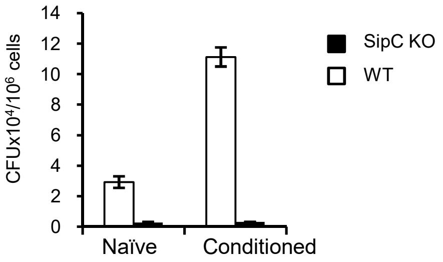

Supplementary Figure S3, Interaction of invasion-poor SipC deficient derivative of *S*. Typhimurium with Hela does not result in increased invasion ability of this pathogen. Hela cells were infected for 1 h at 37˚C with WT and SipC deficient derivative of *S*. Typhimurium (MOI 10),which had been cultured in cell culture medium or conditioned with Hela for 1 h. Extracellular bacteria were washed off and intracellular bacterial load was determined by plating cell lysates on SS agar plates. CFUs were determined after overnight incubation at 37ºC. Data is representative of two independent experiments.

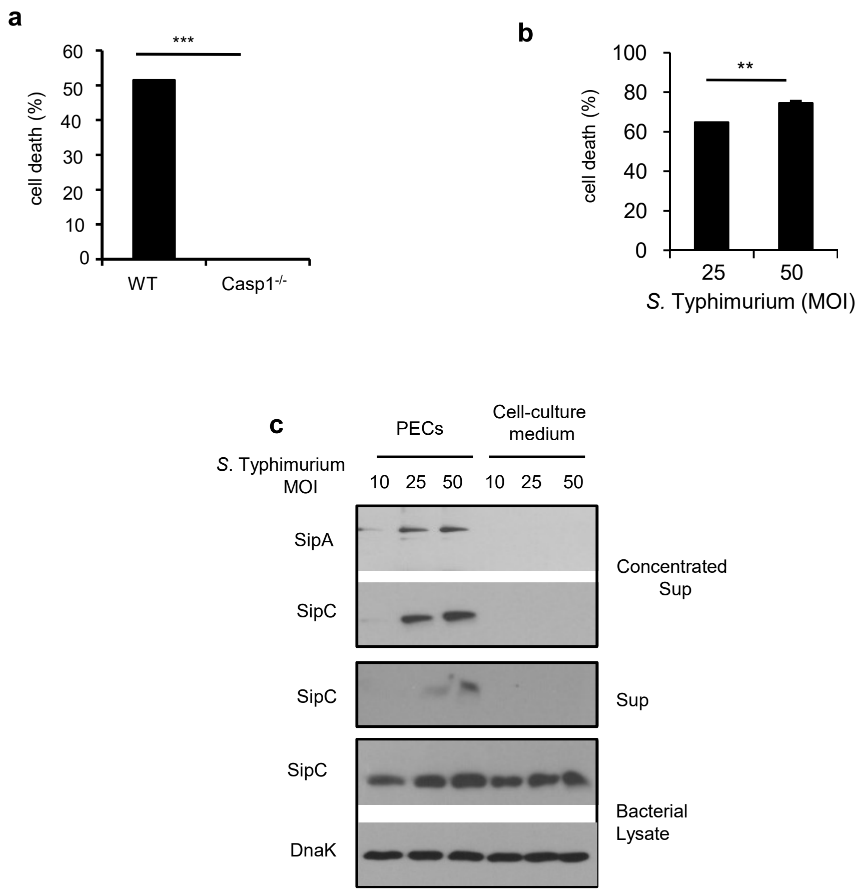

Supplementary Figure S4, Infection of macrophages with *S*. Typhimurium induces caspase-1 mediated cell death, which promotes secretion of Sips from *Salmonella* (**a**) Percentage cell death in iBMDMs infected with *S*. Typhimurium at an MOI of 50. Data representative of three independent experiments is plotted as mean ± SD; ***p< 0.001. (**b**) PECs from C57/BL6 mice were infected with *S.* Typhimurium for 1h at indicated MOIs. Cell death was analyzed by release of lactate dehydrogenase assay. Data representative of three independent experiments is plotted as mean ± SD; **p< 0.01. (**c**) SipA and SipC expression in cell-free supernatants (concentrated by precipitating with TCA) and lysates prepared from bacteria cultured in serum-free cell culture medium or conditioned with PECs at indicated MOIs for 1 h.

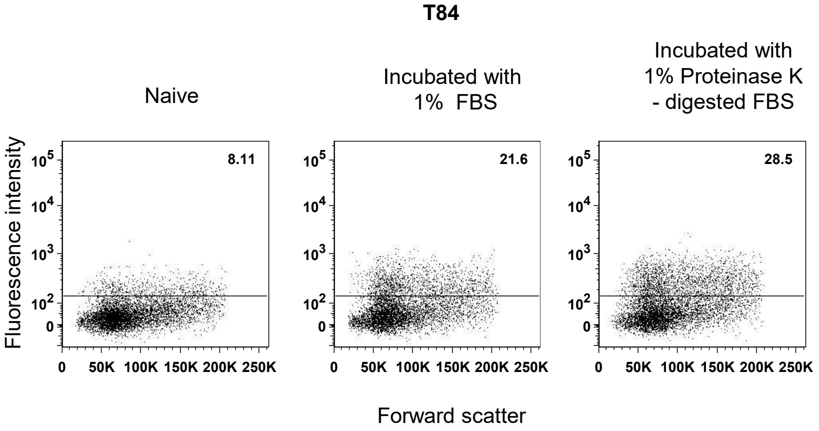

Supplementary Figure S5, Serum-derived Proteinase K – resistant stimulus enhances invasion ability of *S*. Typhimurium. Flow cytometric analysis of T84 cells infected with GFP-labeled *S*. Typhimurium (100 MOI) which had been incubated with indicated concentrations of undigested or Proteinase –K digested serum for 1 h at 37ºC. Numbers indicate percent cells containing GFP-positive bacteria. Data is representative of two independent experiments.

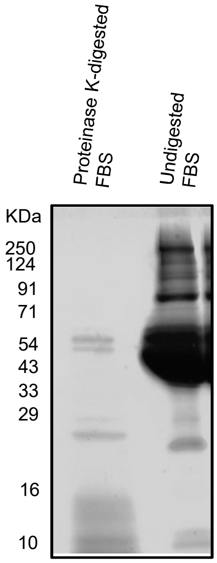

Supplementary Figure S6, Proteinase-K digestion of FBS. Undigested and Proteinase K-digested FBS samples were run in SDS-polyacrylamide gel and stained with coomassie blue.

a

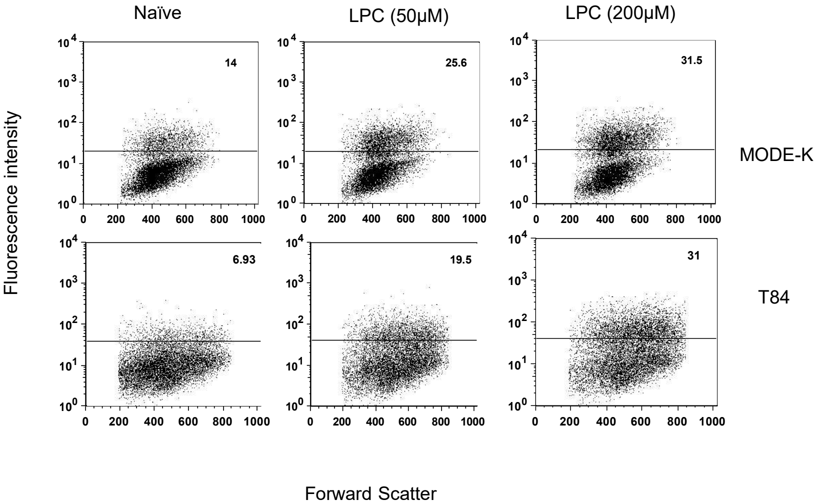

b

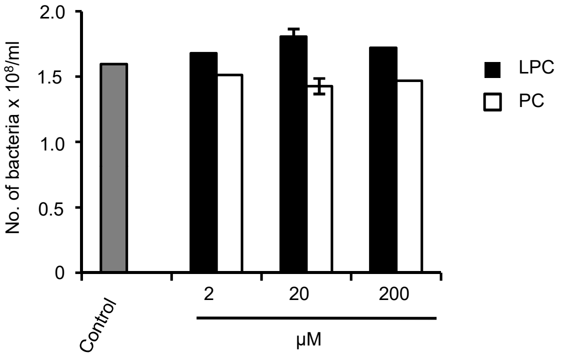

Supplementary Figure S7, LPC-stimulation enhances invasion capability of *Salmonella* without modulating bacterial growth. (**a**) Flow cytometric analysis of MODE-K and T84 cells infected with GFP-labeled *S*. Typhimurium (100 MOI) which had been incubated with indicated concentrations of LPC for 1 h at 37ºC. Numbers indicate percent cells containing GFP positive bacteria. (**b**) Bacterial number was determined by measuring optical density (at 630 nm) of *S*. Typhimurium cultured in serum free medium in the absence (control) or presence of different concentrations of LPC or PC for 1 h at 37ºC. Data representative of two independent experiments is plotted as mean ± SD.

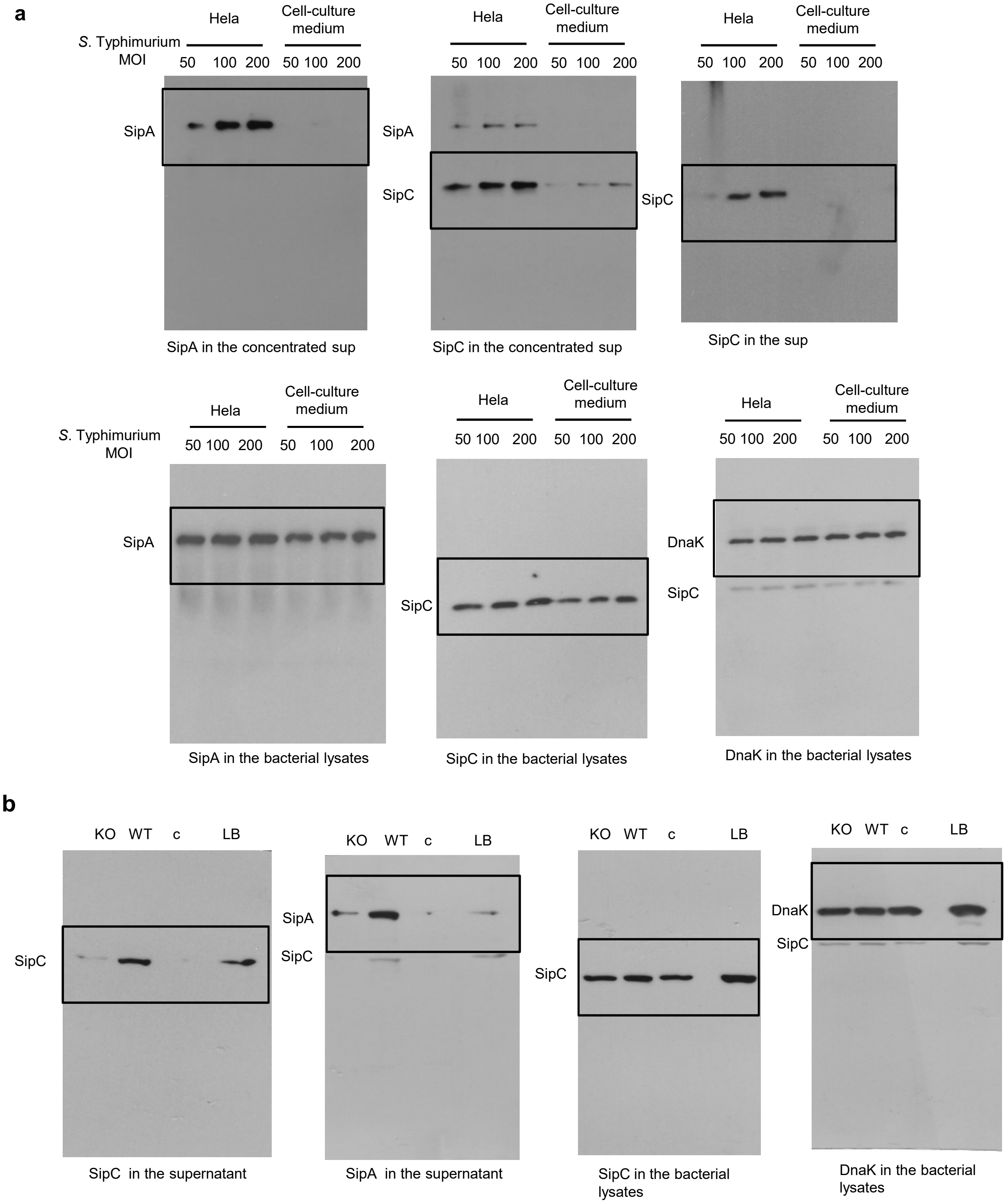

Supplementary figure S8 Full-length immunoblots from main figures. Blots from main figs.1a (**a**) and 1i (**b**) probed with SipC,SipA and DnaK antibodies. Cropped regions used in main figures are indicated by boxes.

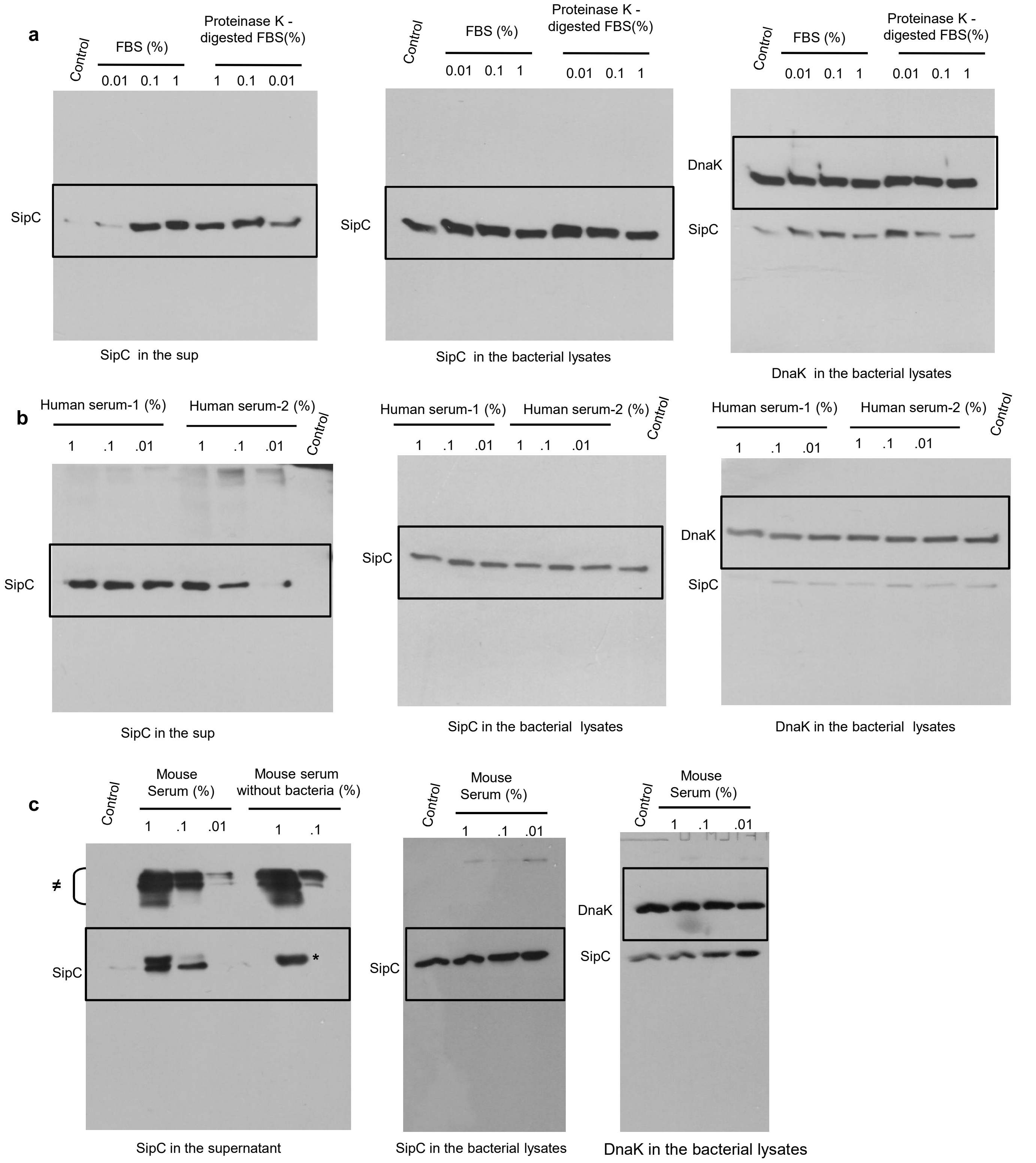

Supplementary figure S9 Full-length immunoblots from main figures. Blots from main fig 2a (**a**) and 2c (**b** and **c**) probed with SipC and DnaK antibodies. ≠ and * are Ig bands and a nonspecific band respectively obtained upon incubation of nitrocellulose membrane with HRP-labeled anti-mouse Ig antibody. Cropped regions used in main figures are indicated by boxes.

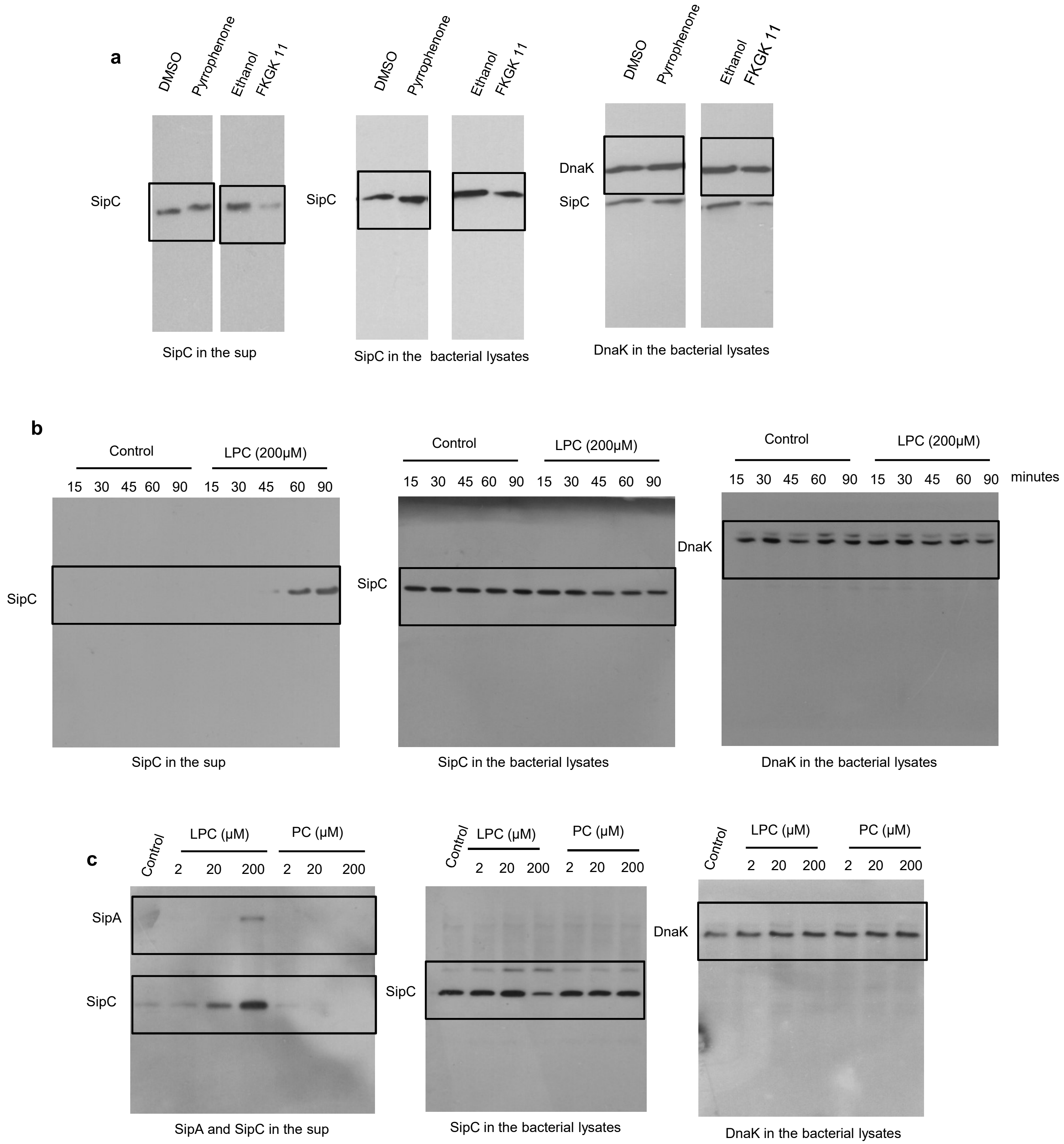

Supplementary figure S10, Full-length immunoblots from main figures. Blots from main figs. 3a (**a**), 3c (**b**) and 3d (**c**) probed with SipC, SipA and DnaK antibodies. Cropped regions used in main figures are indicated by boxes.

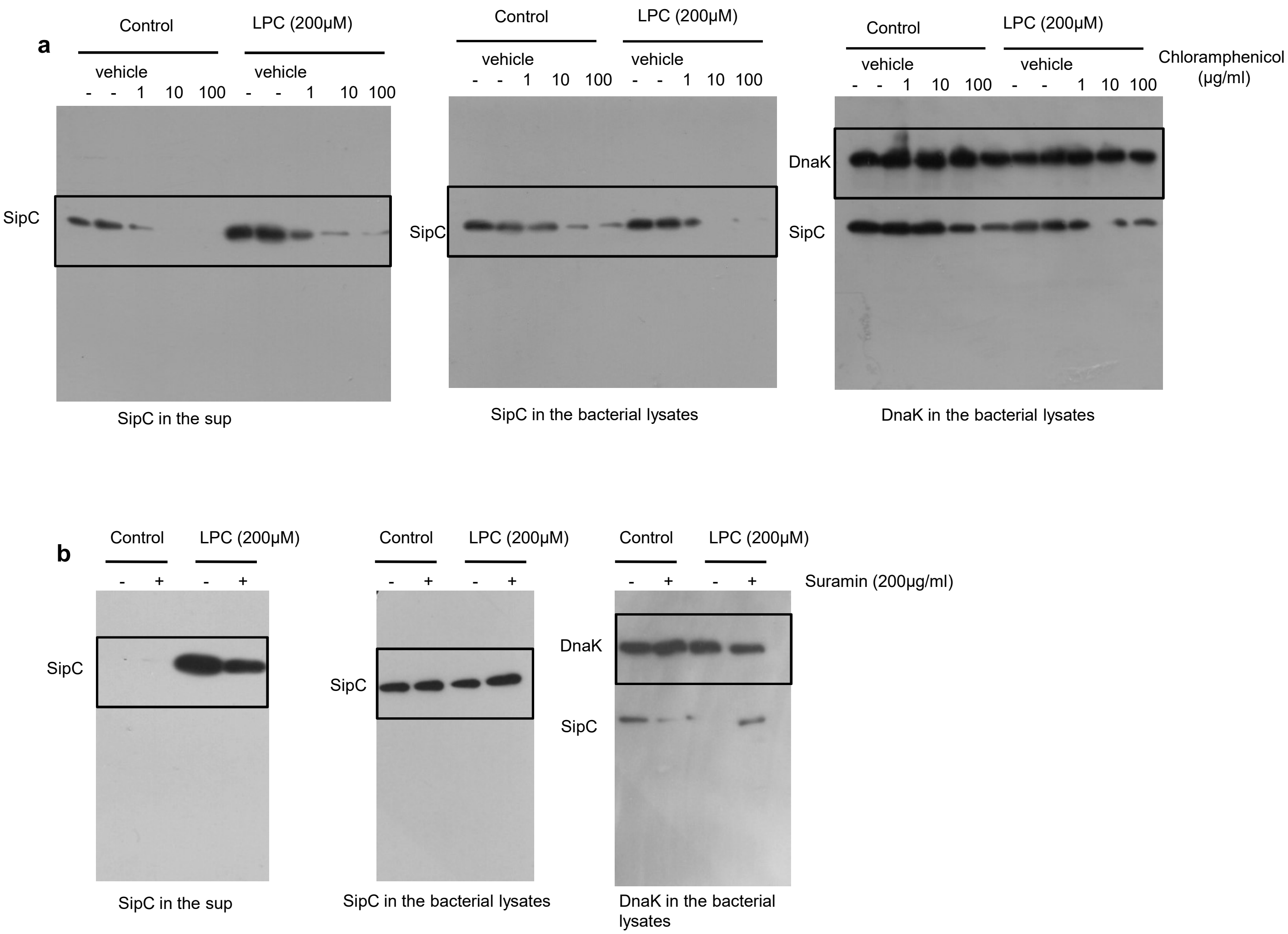

Supplementary figure S11, Full-length immunoblots from main figures. Blots from main figs. 3e (**a**) and 3f (**b**) probed with SipC and DnaK antibodies. Cropped regions used in main figures are indicated by boxes.

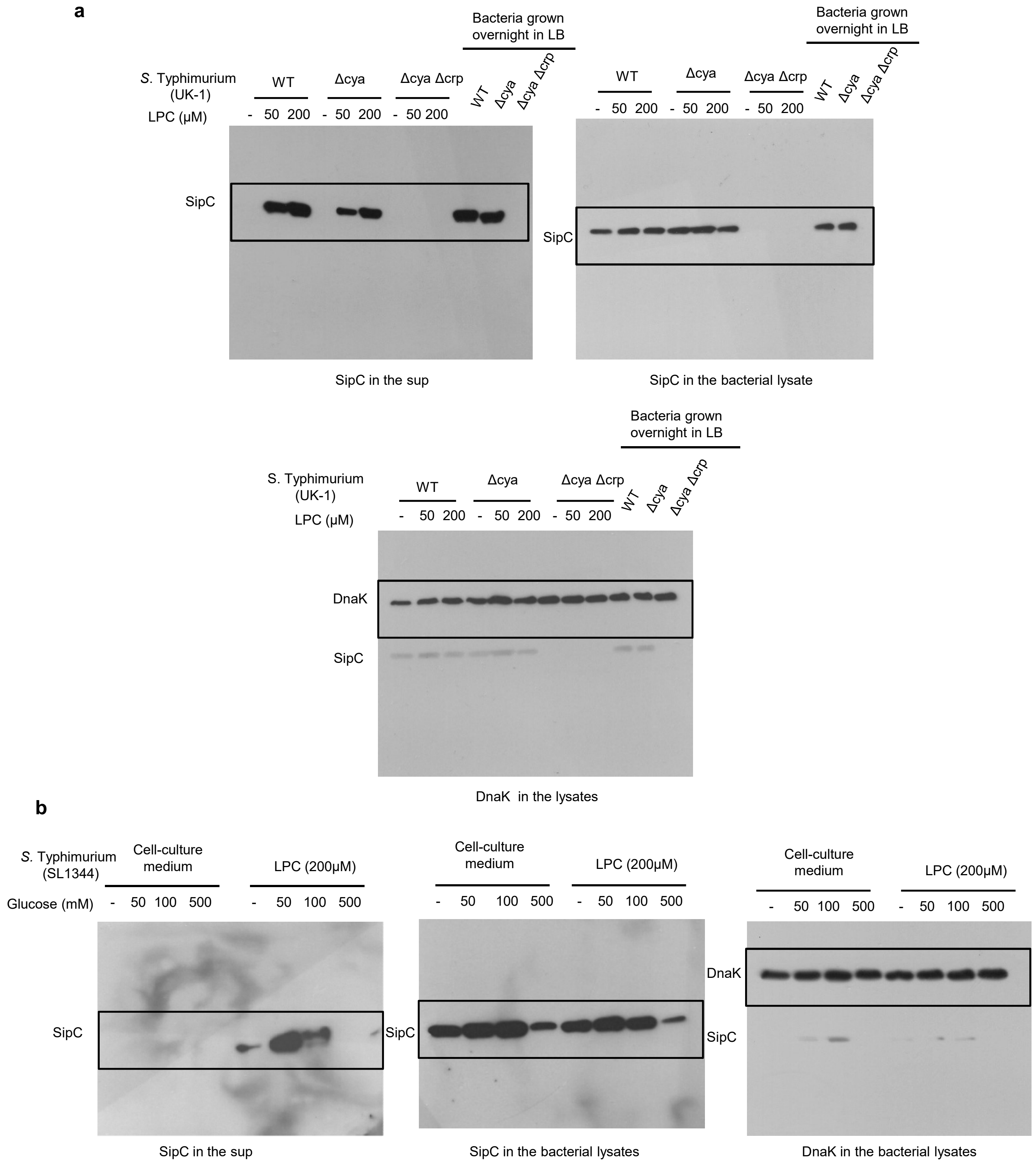

Supplementary figure S12 Full-length immunoblots from main figures. Blots from main figs. 4a (**a**) and 4b (**b**) probed with SipC and DnaK antibodies. Cropped regions used in main figures are indicated by boxes.

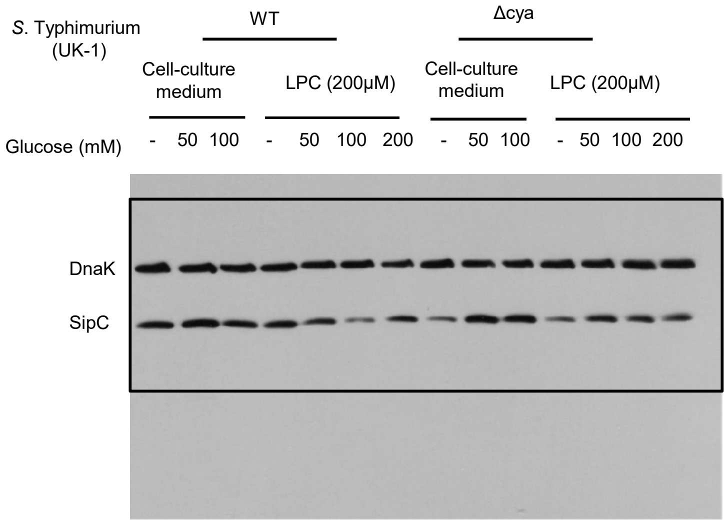

SipC and DnaK in the bacterial lysate

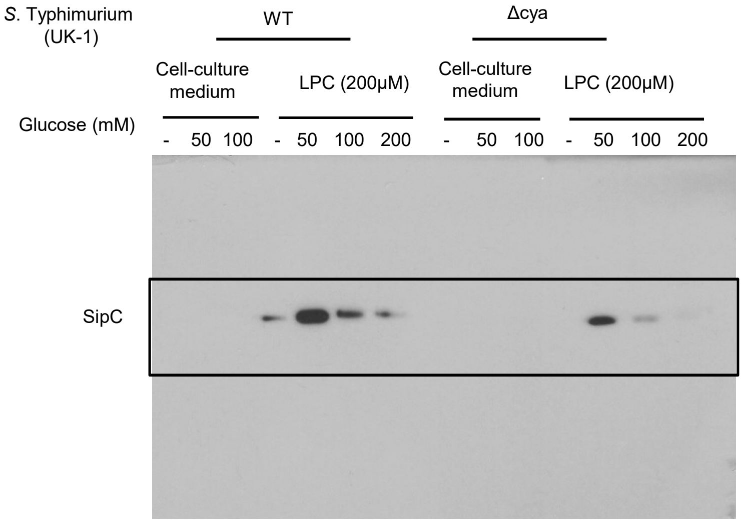

SipC in the sup

Supplementary figure S13 Full-length immunoblots from main figures. Blots from main fig. 4c probed with SipC and DnaK antibodies. Cropped regions used in the main figure are indicated by boxes.

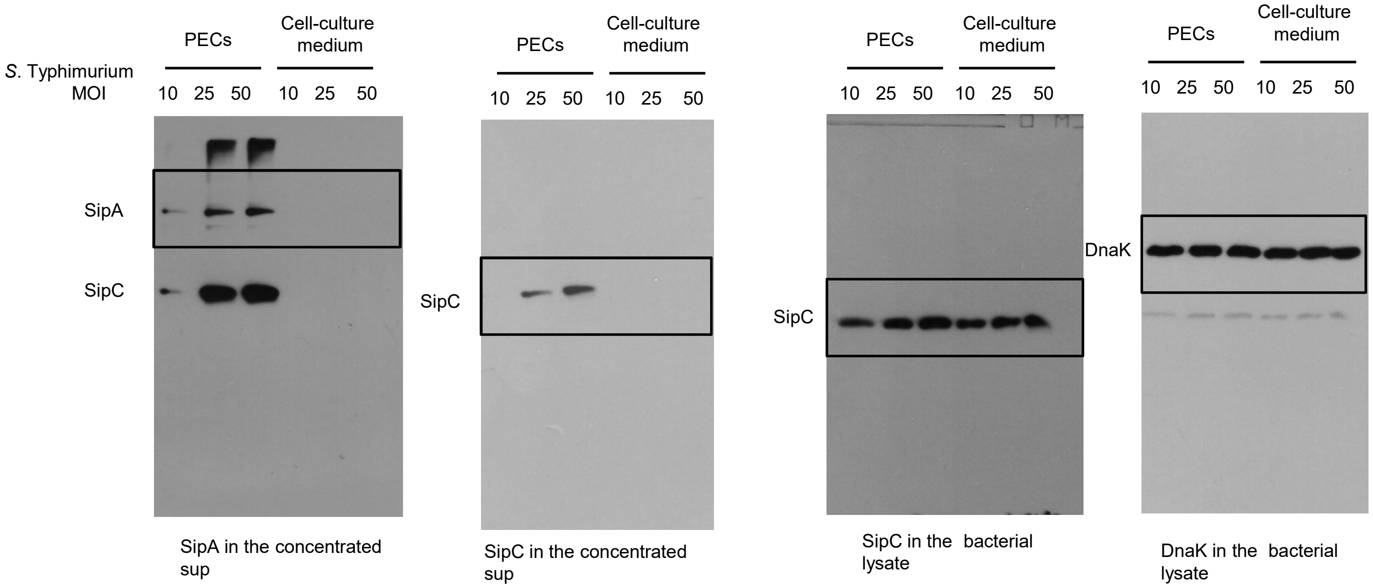

Supplementary figure S14 Full-length immunoblots from Supplementary figure S4c. Blots from Supplementary Fig. S4c probed with SipA, SipC and DnaK antibodies. Cropped regions used in the figure are indicated by boxes.
